# Some Orthopædic Principles Explained
*A lecture delivered at the Institute of Hygiene, April 6, 1921, by W. H. Trethowan, M.B., F.R.C.S.


**Published:** 1921-04-16

**Authors:** 


### THE PERFECT FOOT.

### Some Orthopædic Principles Explained.*

During the whole of the war, and for a considerable period afterwards, Mr. Trethowan was surgeon to one of the great military orthopædic hospitals, and had an extended opportunity for noticing how frequently deformities and disabilities of the feet contributed to the C3 element of the male population who were enrolled in the new army, and also the effect of these foot conditions on active service. At that time we heard of a great number of cases of so-called "trench feet," but when, from the cases thus grouped, those really due to frost-bite were eliminated, the remainder were ordinary mechanical disabilities so common in civil life. Apart from frost-bite, the lecturer has, indeed, never been able to convince himself that such a disease as trench feet ever existed.

### In Civil Life.

It is impossible to find in a civilised adult a perfect foot—one of ideal shape, which will do its work properly and gracefully, and give no trouble. To keep the feet healthy it is necessary to "catch them early," and the most effective work for reform can be carried out by the provision of proper foot-wear for children. Growing children must not be on their feet all day long, and should not be required to follow laborious occupations, entailing heavy lifting.

The commonest trouble met with is flat foot, or dropping of the long arch which lies principally to the inner side of the foot, but in most cases, and practically always in children, the first trouble is not really flat foot—that is, a giving-way of the long arch—but is a falling-in of the foot just below the ankle joint, a condition often referred to as weak ankles, which condition leads to more strain on the inner border of the foot and, later, to the arch itself giving way. The best exercise for flat or weak feet is correct walking, developed until it becomes automatic. Slight turning out of the toes is correct when standing easy, but in walking the feet should be kept parallel.

The essential use of a boot, the lecturer said, is to protect the ligaments which bind the small bones together from over-strain, and, consequently, the boot or shoe should correspond to the shape of the foot. Instead of the shoe fitting the foot, however, it is unfortunately the case that the foot is compelled to adopt the conventional standard shape of the shoemaker, who has his foot narrower than the actual human foot, and produces a pointed-toe portion in which the great toe is cramped, turned outwards, and not permitted to move. So-called square toes are a delusion and a snare, as it is not the part projecting beyond the toes which matters, the important point being that none of the toes must be subjected to side pressure. A good shoe should also have a well-curved and stiffened waist.

### High and Low Heels.

With regard to heels the Cuban is the most satisfactory for general use, while the Louis heel is bad, on account of its curved mechanical shape and inefficient support, and also because it weakens the whole sole of the shoe. In a strong foot the lower, brogue, heel can be used. Children with normal feet do not require much heel. Women, however, favour high heels, and, while there is no doubt that some do so for purely cosmetic reasons, it is certain that the majority of women wear high heels as a response to the relief afforded to the ligaments of the foot.

Women, being hampered by skirts, cannot get such freedom of movement in the hips as men are able to do; and it may be from this cause that women need the more efficient support afforded by heels. The action of the foot in man is not nearly so marked as in women, as men propel themselves with the hip movement of long strides, while women walk more with their feet. It is a universal and traditional idea that the majority of the troubles occurring in the feet are the evil results of the use of heels, but the anatomy and movements of the foot fully show that the strain on the long arch must lessen in proportion as the heel is raised, and this is the experience of the vast majority of women, in spite of medical advice to the contrary.

### A Popular Fallacy.

The most important measure in giving relief in cases of weak or overstrained feet is to raise the height of the heels, and not to lower it, as is nearly always advised. The height of the heel for remedial purposes should not exceed two and a-quarter inches, and in house shoes should not be less than one and a-quarter inches. Naturally the height should do no more than to afford relief. In cases of overstrained feet slippers must be avoided. Heels not less than one and a-quarter or one and a-half inches in height are suitable for general use in healthy feet.

Most ready-made boots, Mr. Trethowan says, fail miserably in the support afforded to the inner border of the foot, and this, together with waists that are thin and unyielding and which bulge downwards after use for two or three weeks, is largely responsible for the inefficient support and breakdown of the foot. Where such an evil arrangement is combined with a flat heel, the shoe is of the worst description. The majority of people habitually wear shoes which are too narrow across the toes and the balls of the toes. Up to a point, just behind the balls of the toes, the snugger and narrower the shoe the better—for a good, tight grip of the instep is very desirable.

### Selecting Shoes.

In selecting shoes, important points to be borne in mind are—the length, the breadth of the soles, the pointing inwards of the toe portion or straight inner border, and the roominess of the toe portion of the upper. Pressure effects begin very early in children, and a single pair of ill-fitting shoes may be the cause of a considerable degree of permanent deformity. Socks and stockings are of some importance also, and are often too short and narrow, and made of unyielding materials. Socks, in order not to have any deforming effects, should be large, not pointed in the toes, and made of material that will readily expand.

* A lecture delivered at the Institute of Hygiene, April 6, 1921, by W. H. Trethowan, M.B., F.R.C.S.

